# Effectiveness of Auricular Acupuncture for Insomnia: An Overview of Systematic Reviews

**DOI:** 10.1155/2020/6920902

**Published:** 2020-05-13

**Authors:** Jinke Huang, Min Shen, Xiaohui Qin, Yong Huang

**Affiliations:** ^1^The Second Clinical Medical College of Guangzhou University of Chinese Medicine, Guangzhou, Guangdong Province 510120, China; ^2^Department of Neurology, The Second Affiliated Hospital of Guangzhou University of Chinese Medicine (Guangdong Provincial Hospital of Chinese Medicine), Guangzhou, Guangdong Province 510120, China; ^3^School of Traditional Chinese Medicine, Southern Medical University, Guangzhou, Guangdong Province 510515, China

## Abstract

**Objectives:**

The effectiveness of auricular acupuncture (AA) for insomnia is far from uniform. The aim of this overview was to summarize and critically evaluate the evidence from systematic reviews (SRs)/meta-analysis (MAs) and provide an overall verdict about the therapeutic value of AA for insomnia.

**Methods:**

A search of relevant literature for SRs/MAs was performed on major medical databases. The methodological quality was assessed using the Assessing the Methodological Quality of Systematic Reviews 2 (AMSTAR-2) and the evidence quality was assessed using the Grading of Recommendations, Assessment, Development and Evaluation (GRADE).

**Results:**

Seven SRs/MAs were deemed eligible for the present overview. According to the evaluation results of AMSTAR-2, the methodological quality of all included SRs/MAs was critically low. Consistent methodological deficiencies were item 2 (the lack of a protocol), item 4 (the lack of a specific search strategy), item 7 (the lack of a list of excluded studies), and item 15 (the lack of an assessment of publication bias). For GRADE, of the 17 outcomes, only 1 (5.9%) was rated of high-quality, 4 (23.5%) were rated of moderate-quality, and the remaining 12 (70.6%) were rated of low-or critically low-quality. Descriptive analysis of the outcomes reveals a positive effect of AA for insomnia.

**Conclusions:**

AA may be beneficial for insomnia, but the evidence is plagued by important limitations, e.g., the poor quality of SRs/MAs and primary studies.

## 1. Introduction

Insomnia is a common disease caused by multiple environmental and psychological factors, which may lead to anxiety, depression, immune functioning, cardiovascular disease, and even suicide, seriously damaging the physical and mental health of patients [1]. Worldwide, around 15% to 30% of adults and 10% to 23% of adolescents have different degrees of insomnia [2]. Furthermore, the incidence of insomnia increases with age and is more likely to affect females [3]. Therefore, more researchers were beginning to notice insomnia [4]. Currently, the main treatments recommended for insomnia are cognitive-behavioral therapy, pharmacotherapy, and exercise therapy. In spite of the continuous progress in the pharmacotherapy of insomnia, the use of complementary and alternative therapies as a sole treatment or combined with pharmacotherapy is common.

Among the complementary and alternative therapies, acupuncture has been regarded as a promising method to treat insomnia, and it has been recommended as a complementary treatment option for insomnia in the latest guidelines by the China Sleep Research Association [5]. Increasing evidence shows that auricular acupuncture (AA) can improve the sleep quality of insomnia. However, evidence quality has not been evaluated. Therefore, this study hopes to evaluate the effectiveness of AA on insomnia and provide new evidence for clinical application.

## 2. Methods

### 2.1. Inclusion Criteria

#### 2.1.1. Type of Studies

The studies reviewed are systematic reviews (SRs)/meta-analyses (MAs) based on randomized controlled trials (RCTs) of AA for insomnia. The languages of the SRs/MAs are limited to Chinese and English. Non-SRs/MAs, a series of case reports, and other types of studies were excluded.

#### 2.1.2. Interventions

Studies that involved the use of AA or AA plus conventional medication (CM) in the intervention group were included. The following treatments were used in the control group: CM, placebo, cognitive-behavioral therapy, pharmacotherapy, exercise therapy, or other nondrug therapies.

#### 2.1.3. Outcome

Effective rate, sleep parameters, sleep efficiency, scales, or index for sleep quality evaluation, adverse effect.

### 2.2. Search Strategy

The following five electronic databases were systematically retrieved from their inception to March 2020: Web of Science, The Cochrane Library, PubMed, Embase, and Sino-Med. The following keywords were used: dyssomnia, insomnia, auricular acupuncture, systematic review, and meta-analysis. The search strategy for the PubMed database is presented in Table 1, and it would be adjusted for each database.

### 2.3. Data Extraction

Two reviewers (JK-H and XH-Q) screened titles and abstracts according to the eligibility criteria. Studies that met the inclusion criteria were evaluated with full text. For the articles finally included, two reviewers independently extracted the following information: first author, year of the publication, simple, intervention, quality assessment, outcomes, and main results. Any disagreement that occurred on study inclusion or data extraction between them was resolved by discussion or by referral to a third reviewer for a final decision.

### 2.4. Quality Assessment

Two reviewers (JK-H and XH-Q) assessed the methodological quality of SRs/MAs with Assessing the Methodological Quality of Systematic Reviews 2 (AMSTAR-2) [6]. The checklist consists of 16 items answerable by a yes, partial yes, or no responses. After interpreting weaknesses detected in critical and noncritical items, the methodological quality of SRs/MAs was judged as very low, low, moderate, or high.

Two reviewers (JK-H and XH-Q) assessed the evidence quality for each outcome with Grades of Recommendation, Assessment, Development, and Evaluation (GRADE) [7]. The factors that potentially could affect the reliability of the given effect estimate included limitations (that is, a risk of bias of RCTs), inconsistency, indirectness, imprecision, and publication bias [8]. After considering the factors in terms of the above mentioned, the evidence quality of each outcome was judged as very low, low, moderate, or high. Any disagreement that occurred on the quality assessment between them was resolved by discussion or by referral to a third reviewer for a final decision.

## 3. Results

### 3.1. Literature Selection

**Figure 1:**
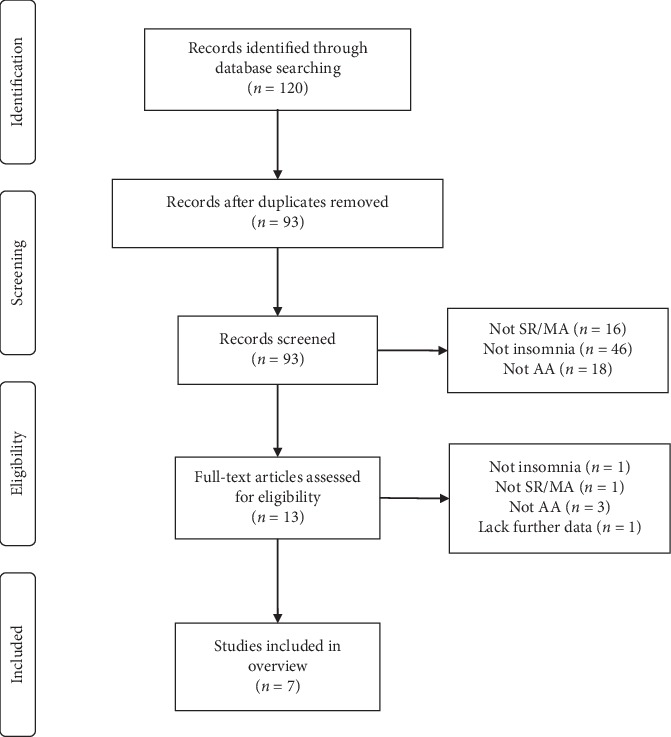
A flowchart of the literature search and selection process.

A database search identified 120 publications, of which 107 were excluded after the title and/or abstract screening, and 13 were subjected to eligibility criteria apply. After review of the complete manuscripts, 7 articles [9–15] met the eligibility criteria, and 6 were excluded (1 was not regarding insomnia, 1 was not SR/MA, 3 were not regarding AA, and 1 lacked further data).  Figure 1 shows the diagram with the selection procedure.

### 3.2. Study Characteristics

The characteristics of the 7 SRs/MAs included in our final analysis are summarized in Table 2. The included SRs/MAs were published from 2007 to 2019, 3 were written in Chinese [9–11], and the remaining 4 [12–15] were written in English. Each SRs/MAs comprised 2 to 40 trails. The subjects included in each SRs/MAs were 459 to 4115. Of the 7 SRs/MAs, 85.7% of the SR authors were from China (*n* = 6), 14.3% from Korea (*n* = 1). Interventions in the treatment group were mainly AA, and the control group were mainly placebo and CM. Different versions of quality assessment scales were used as the methodological quality assessment tools, 5 [10–13, 15] used Cochrane risk of bias criteria, and the remaining 2 [9, 14] used Jadad.

### 3.3. Methodological Quality

According to the evaluation criteria of AMSTAR-2, since all SRs/MAs had more than one critical weakness, their methodological quality was regarded as critically low. Details of assessment results are given in Table 3.

### 3.4. Evidence Quality

Seventeen outcomes related to the effectiveness of AA for insomnia were included in seven SRs/MAs. According to the evaluation criteria of GRADE, the evidence quality ranged between high and critically low, only 1 outcome was rated of high-quality, and 4 were rated of moderate-quality. The most common degradation factors were the risk of bias within the original trials, inconsistency, imprecision, and the possibility of publication bias (Table 4).

### 3.5. Meta-Analyses Outcomes of Intervention

#### 3.5.1. AA versus Placebo

Four reviews [11–14] compared the effects of AA with placebo. Three out of the 4 reviews [11–13] revealed that there was a significantly greater reduction in PSQI scores in the AA group than in the placebo. Electroencephalogram, polysomnogram, and wrist actigraphy were adopted in 2 reviews [11, 12] to record sleep status and the pooled results showed sleep onset latency was shortened in the AA group compared to the placebo. Two reviews [11, 12] suggested a reduction in the number of awakenings between AA and placebo, and the difference was statistically significant. One review [12] mentioned sleep time as an effectiveness scale; statistics showed that AA was more likely to prolong total sleep time compared to the placebo group. Furthermore, 2 reviews [12, 14] reported that AA was superior to placebo in sleep efficiency. The results mentioned above suggested that AA was favored over the placebo for treating insomnia.

#### 3.5.2. AA versus CM

Four reviews [9, 10, 12, 15] compared the effects of AA with CM. Three out of the 4 reviews [9, 10, 12] reported the effective rate to AA for insomnia, and the results showed that the AA was superior to CM on insomnia. One review [9] evaluated the therapeutic effect of AA for insomnia using the PSQI score, and the result showed that there was a significantly greater reduction in PSQI score in the AA group than in the CM group. One review [15] mentioned sleep time as an effectiveness scale. Statistics showed that AA was more likely to prolong total sleep time compared to the CM group. Furthermore, 1 review [15] reported that AA was superior to CM in sleep efficiency. In terms of advent events, results demonstrated that AA caused less frequent and less severe adverse events compared with CM [12]. The results mentioned above suggested that AA was favored over the CM for treating insomnia with less adverse events.

## 4. Discussion

As an adjunct to acupuncture, AA is based on the idea that the outer ear has a somatotropin map with an inverted fetus pattern, and each part of the auricle is corresponding to a specific part of the human body or organ [16]. By stimulating specific auricular points, AA may have positive effects by rebalancing the central nervous system and alleviating multiple pathological conditions. Increasing evidence shows that AA may be used as adjunctive therapy in insomnia, reducing the use of CM, and minimizing the potential adverse effects. The present overview aimed to collect and rate the scientific evidence from the SRs/MAs on the effect of AA for insomnia.

### 4.1. Summary of Main Results

In our overview, evidence on the efficacy and safety of AA on insomnia was synthesized from 7 SRs/MAs. Overall, the existing evidence suggested that AA was more effective than a placebo or CM for treating insomnia. Regarding safety, no serious adverse effects were associated with AA. However, the number of trials and the total sample size was too small; the overall methodological quality and the reporting of data were generally poor. These factors led the authors of most SRs/MAs not to draw firm conclusions. Furthermore, according to the assessment results of AMSTAR-2 and GRADE, the methodological quality and the evidence quality of the included SRs/MAs are generally unsatisfied, indicating that the results of SRs/MAs may be very different from the real situation. Hence, further studies with the improved methodological design are needed to accurately determine the efficacy and safety of these modalities for insomnia.

### 4.2. Quality Summary of Included SRs/MAs

According to the assessment results of AMSTAR-2, the methodological quality of all included SRs/MAs was rated as critically low. Consistent methodological deficiencies are as follows: item 2 (the lack of a protocol), which may produce a larger adjustment of the study process than expected, affecting the rigor of the SR/MA. It is noted that registering a protocol in advance can help to facilitate processing transparency and to avoid the risk of bias in methodology [17]. Item 4 (the lack of a specific search strategy) may generate publication bias. A complete and transparent search process is the best way to avoid publication bias. Item 7 (the lack of a list of excluded studies) may affect the authenticity of the results, providing a list of potentially relevant studies that do not meet the inclusion criteria and account the reason for exclusion is an indispensable part of a high-quality SR/MA. Item 15 (the lack of an assessment of publication bias) may destroy the authenticity of the conclusion. These findings indicating that there is much room for addressing the methodological quality during the SR/MA process. High-quality SRs/MAs will be helpful in providing scientific evidence for clinicians, patients, and other evidence users [18]. Therefore, researchers should pay attention to comply with the requirements of the relevant items of the AMSTAR-2 and strictly controlling the methodological quality of the studies.

According to the assessment results of GRADE, the evidence quality ranged between high and critically low, only 1 outcome was rated of high-quality, 4 were rated of moderate-quality, and the remaining 12 were rated of low- or critically low-quality. The lower the quality is the more likely further research would change our confidence in the estimates, and the estimates themselves [19]. Therefore, caution should be warranted when recommending AA as an alternative treatment for treating insomnia. The most common degradation factors were the risk of bias within the original trials, inconsistency, imprecision, and the possibility of publication bias. Of the majority of included RCTs within these SRs/MAs, the methodological quality of them was of low quality. Consistent methodological deficiencies are as follows: RCTs were described as randomized without providing the method of random sequence generation; most RCTs did not explicitly state that treatment allocation was concealed; blinding of subjects and physicians for most RCTs failed, though they should have been blinded ideally. These findings indicate that there is much room for addressing the methodological quality during the RCT process. High-quality RCTs with large sample sizes should be the focus of future research.

### 4.3. Strengths and Limitations

As an overview of AA for treating insomnia, our study can provide scientific evidence reference for decision-making in the clinic. Furthermore, the evaluation process of AMSTAR-2 and GRADE showed methodological deficiencies of SRs/MAs and RCTs, which may help to guide future high-quality studies. However, it is also subject to limitations. We included only SRs/MAs published in English and Chinese, so a small group of studies in other languages might have been missed.

## 5. Conclusion

To conclude, AA may be beneficial for insomnia. However, physicians should apply the evidence to make decisions about AA for treating insomnia with caution in clinical practice owing to the generally low methodological quality and evidence quality in the included SRs/MAs.

## Figures and Tables

**Table 1:** Search strategy for the PubMed database.

Query	Search term
# 1	Dyssomnias [Mesh]
# 2	Dyssomnias [Title/Abstract] OR insomnia [Title/Abstract] OR sleep disorders [Title/Abstract] OR sleep initiation and maintenance disorders [Title/Abstract] OR wakefullness [Title/Abstract] OR somnipathy [Title/Abstract]
# 3	#1 OR #2
# 4	Aricular acupuncture [Mesh] OR ear acupuncture[Mesh]
# 5	Auricular acupuncture [Title/Abstract] OR ear acupuncture [Title/Abstract] OR auricular acupressure[Title/Abstract] OR auricular therapy[Title/Abstract] OR acupressur^*∗*^ [Title/Abstract] OR auricular [Title/Abstract] OR auriculotherap^*∗*^ [Title/Abstract] OR auriculotherapy [Title/Abstract] OR auricular needle [Title/Abstract] OR Otoneedle [Title/Abstract]
# 6	#4 OR #5
# 7	Meta-analysis as Topic [Mesh]
# 8	Systematic review[Title/Abstract] OR meta-analysis [Title/Abstract] OR meta-analyses [Title/Abstract]
# 9	#7 OR #8
# 10	#3 AND #6 AND #9

**Table 2:** Characteristics of the included reviews.

Studies	Country	Trials (subjects)	Treatment intervention	Control intervention	Quality assessment	Results
Li [9]	China	6 (459)	AA	CM	Jadad	AA can reduce the PSQI score and improve the sleep of patients with insomnia.

Yang [10]	China	7 (939)	AA	CM	Cochrane criteria	AA has a certain effect on insomnia and improves patients' sleep quality.

Tan [11]	China	8 (894)	AA	CM; placebo	Cochrane criteria	AA can effectively improve sleep quality, but due to the low evidence quality, cautious attitude should be taken on this conclusion.

Lan [12]	China	15 (1381)	AA	CM; placebo	Cochrane criteria	Statistical analyses of the outcomes reveal a positive effect of AA for insomnia.

Yeung [13]	China	40 (4115)	AA	CM; placebo	Cochrane criteria	Owing to the methodological limitations of the studies and equivocal results, the current evidence does not allow a clear conclusion on the benefits of AA for insomnia.

Lee [14]	Korean	10 (1540)	AA	CM; placebo	Jadad	Because of the paucity and of the poor quality of the data, the evidence for the effectiveness of AA for the symptomatic treatment of insomnia is limited.

Chen [15]	China	6 (673)	AA	CM; placebo	Cochrane criteria	AA appears to be effective in treating insomnia. Further clinical trials with higher design quality, longer duration of treatment, and longer follow-up should be conducted.

AA: auricular acupuncture; CM: conventional medication.

**Table 3:** Result of the AMSTAR-2 assessments.

Reviews	AMSTAR-2																Quality
	Q1	Q2	Q3	Q4	Q5	Q6	Q7	Q8	Q9	Q10	Q11	Q12	Q13	Q14	Q15	Q16	
Li et al. [9]	Y	PY	Y	PY	Y	Y	N	Y	Y	N	Y	Y	Y	Y	N	N	CL
Yang et al. [10]	Y	PY	Y	PY	Y	Y	N	Y	Y	N	Y	Y	Y	Y	N	N	CL
Tan et al. [11]	Y	PY	Y	PY	Y	Y	N	Y	Y	Y	Y	Y	Y	Y	N	Y	CL
Lan et al. [12]	Y	PY	Y	Y	Y	Y	N	Y	Y	Y	Y	Y	Y	Y	Y	Y	CL
Yeung et al. [13]	Y	PY	Y	Y	Y	Y	N	Y	Y	Y	Y	Y	Y	Y	N	Y	CL
Lee et al. [14]	Y	PY	Y	PY	Y	Y	N	Y	Y	Y	Y	Y	Y	Y	N	Y	CL
Chen et al. [15]	Y	PY	Y	PY	Y	Y	N	Y	Y	Y	Y	Y	Y	Y	N	Y	CL

Y: yes; PY: partial yes; N: no; CL: critically low; L: low; H: high.

**Table 4:** Evidences quality of SRs/MAs.

Reviews	Interventions	Outcomes	Limitations	Inconsistency	Indirectness	Imprecision	Publication bias	Quality
Li et al. [9]	AA versus CM	PSQI score	−1	−1	0	0	−1	L
		Effective rate	−1	0	0	0	−1	CL
Yang et al. [10]	AA versus CM	Effective rate	−1	−1	0	0	0	L
Tan et al. [11]	AA versus placebo	PSQI score	−1	0	0	0	0	M
		Sleep onset latency	−1	−1	0	−1	−1	CL
		Number of awakenings	−1	0	0	−1	−1	CL
Lan et al. [12]	AA versus placebo	Sleep time	−1	0	0	0	−1	L
		Sleep efficiency	−1	0	0	−1	−1	CL
		PSQI score	−1	0	0	0	0	M
		Number of awakenings	−1	−1	0	−1	−1	CL
		Sleep onset latency	−1	0	0	−1	−1	CL
	AA versus CM	Effective rate	−1	0	0	0	0	M
		Adverse effects	−1	0	0	0	0	M
Yeung et al. [13]	AA versus placebo	PSQI score	−1	−1	0	−1	−1	CL
Lee et al. [14]	AA versus placebo	Sleep efficiency	0	0	0	0	0	H
Chen et al. [15]	AA versus CM	Sleep efficiency	−1	0	0	−1	−1	CL
		Sleep time	−1	−1	0	−1	−1	CL

−1: downgrade; 0: not downgrade; CL: critically low; L: low; M: moderate; H: high; AA: auricular acupuncture; CM: conventional medication; PSQI: Pittsburgh Sleep Quality Index.

## Data Availability

All data generated or analyzed during this study are included in this published article.

## Abbreviations

AA: Auricular acupuncture
SR: Systematic review
MA: Meta-analysis
AMSTAR-2: Assessing the methodological quality of systematic reviews 2
GRADE: Grading of recommendations, assessment, development, and evaluation
RCTs: Randomized clinical trials
PSQI: Pittsburgh Sleep Quality Index.
